# Anti‐Amyloid Treatments for Alzheimer’s Disease: Patients’ and Families’ Perception and Experience with Drug Consultation

**DOI:** 10.1002/alz.089893

**Published:** 2025-01-09

**Authors:** Allison Lapins, Kate Lucca, Darby Morhardt, Sarah Welch

**Affiliations:** ^1^ Mesulam Center for Cognitive Neurology and Alzheimer’s Disease, Northwestern University Feinberg School of Medicine, Chicago, IL USA; ^2^ Beuhler Center for Health Policy and Economics, Northwestern University Feinberg School of Medicine, Chicago, IL USA

## Abstract

**Background:**

Drug consultations for the new anti‐amyloid therapies for Alzheimer’s Disease are currently taking place at the Northwestern Neurobehavior and Memory Clinic with potentially eligible patients and their families. Study aims are to: 1) measure patient and family perception, barriers and concerns of the drug treatment following a formal consultation or informal discussion with a neurologist, and 2) gather data on the patient and family experience with the drug consultation as a method for making an informed decision.

**Methods:**

Participants who are potentially eligible for anti‐amyloid therapies and have a drug consultation with their behavioral neurologist will be asked to participate in our study survey. Patients who are potentially eligible for anti‐amyloid therapies must be in the early stages of the disease and have biomarker evidence of Alzheimer’s Disease pathology. Interested participants complete the survey over the phone with a study team member. The survey asks questions aimed to measure interest in starting this treatment (before and after consultation), concerns and barriers surrounding the treatment, as well as overall experience with the drug consultation.

**Result:**

This study is underway with data collection and will have preliminary results to share in the coming months.

**Conclusion:**

We expect that drug consultations with patients and families on the new Alzheimer Disease therapies will help to identify concerns, understanding of the risks and benefits and overall impact of taking these newly available treatments.

